# Epileptic syndromes: From clinic to genetic

**Published:** 2015-01-05

**Authors:** Abbas Tafakhori, Vajiheh Aghamollaii, Sara Faghihi-Kashani, Payam Sarraf, Laleh Habibi

**Affiliations:** 1 Department of Neurology, School of Medicine, Imam Khomeini Hospital AND Iranian Center of Neurological Research, Tehran University of Medical Sciences, Tehran, Iran; 2 Department of Neurology, School of Medicine, Roozbeh Hospital AND Iranian Center of Neurological Research, Tehran University of Medical Sciences, Tehran, Iran; 3 Department of Neurology, School of Medicine, Tehran University of Medical Sciences, Tehran, Iran; 4 Department of Medical Genetics, School of Medicine, Tehran University of Medical Sciences, Tehran, Iran

**Keywords:** Epilepsy, Genetic, Inheritance, Chromosomal Abnormalities, Mutation

## Abstract

Epilepsy is one of the most common neurological disorders. Studies have demonstrated that genetic factors have a strong role in etiology of epilepsy. Mutations in genes encoding ion channels, neurotransmitters and other proteins involved in the neuronal biology have been recognized in different types of this disease. Moreover, some chromosomal aberration including ring chromosomes will result in epilepsy. In this review, we intend to highlight the role of molecular genetic in etiology of epilepsy syndromes, inspect the most recent classification of International League against Epilepsy and discuss the role of genetic counseling and genetic testing in management of epilepsy syndromes. Furthermore, we emphasize on collaboration of neurologists and geneticists to improve diagnosis and management.

## Introduction

***Epilepsy***

*Definition*

“Epilepsy” is derived from the Latin term meaning, “to be attacked.” In medicine, epilepsy is defined as recurring episodes of seizures due to excessive and abnormal synchronous neural activity of the cerebral cortex, which could be induced by cellular or molecular defects in cerebral tissues.^1^ In cases of an altered endocrine or metabolic state, it would be categorized as structural/metabolic epilepsy. However, on occasions the underlying disorder could not be recognized, and it would be classified under unknown category. Epilepsies attributed to known genetic disorders are classified as genetic epilepsies^2^ (refer to the next section).

Annual incidence of seizures in the general population is estimated to be 61/100,000 persons^3^ with higher occurrence in both extremes of life.^4^

Clinical diagnosis of epilepsy is carried out mainly by evaluation of patient’s detailed history. Positive electroencephalogram (EEG) results are supportive for confirmation of epilepsy. Nevertheless, negative findings might not exclude the diagnosis of epilepsy.^5^^,^^6^ Clinical presentation of epilepsy may easily be mistaken with conditions mimicking seizure’s features, including hypoglycemia, sleep disorders, migraines, transient ischemic attacks and transient global amnesia.^7^^,^^8^

*Classification*

Based on the 2010 report of International League Against Epilepsy (ILAE),^9^ the etiology of epileptic seizures is divided into three major classes as discussed below.

Genetic epilepsy: this category (previously known as idiopathic) implies epileptic disorders that are a direct consequence of either known single gene defects or complex inheritances in which the epilepsy is the essential symptom. Nonetheless, the contribution of environmental factors in disease expression cannot be disregarded.^2^^,^^9^ The recent alternation of “idiopathic” to “Genetic” has the advantage of highlighting the genetic predisposition, and it no longer conflate other concepts (e.g. prognosis). Most cases display clinical features during childhood or adolescence. Although some suffers from a variety of subtle cognitive and behavioral challenges, the affected patients may have normal intelligence, and EEG might also show generalized discharges.^9^^,^^10^ Genetic epilepsy is further divided into generalized and partial epilepsy. Childhood absence epilepsy, juvenile myoclonic epilepsy and epilepsy with grand-mal seizures on awakening are examples of genetic generalized and benign focal epilepsy of children is an instance of partial genetic epilepsy.^2^^,^^9^

Structural/metabolic epilepsy: Epilepsies classified under this category (previously known as symptomatic) require specific structural or metabolic defects that have been demonstrated to be associated with considerably higher risk of epilepsy. Genetic abnormalities, including mutations and chromosomal abnormalities (e.g. tuberous sclerosis) might be the origin of this category of epilepsy with a particular metabolic or structural disorder inserted between genetic defect and occurrence of epilepsy.^9^

Unknown epilepsy: this category has replaced the previous classification known as cryptogenic. It should be noted that this category contains epileptic disorders, which the underlying cause is not yet determined and could be a consequence of a genetic or separate defect.^9^^,^^11^ Considering the enhancement of genetic methods and improved neuroimaging techniques, the prevalence of unknown epilepsy is decreasing.^11^

***Role of genetic in etiology of epilepsy***

Since establishment of Mendelian Inheritance laws in 1865, modern science launched numerous investigations to discover the role of genetics in pathology of human diseases. The recognized scholarly debate of nature versus nurture, a popular concept in epilepsy disorders, have influenced research agendas for a century and many pioneers tried to unveil this mystery by studying monozygote (MZ) vs. dizygote twins (DZ).^12^^-^^15^ These approaches provide an opportunity to decompose the variables into genetic and environmental factors. Identical twins share about 100% of their genes, while fraternal twins share nearly 50%, and both share many aspects of the environment by virtue of being born in the same place and time. Detection of a particular trait to be substantially more common in MZ twins implicates the importance and strength of genetic determinants in expression of the specific trait.^16^

Concordance of epilepsy has been estimated 62% in MZ pairs compared with 18% in DZ twins.^13^ Large twin population studies suggest a higher rate of epilepsy syndromes in MZ pairs,^13^^-^^15^ specifically generalized epilepsy.^13^ These findings propose the involvement of syndrome-specific genetic determinants in pathology of this group of disorders.

It has been estimated that genetic epilepsy affects 0.3-0.5% of general population.^1^^,^^17^ Children of one parent with genetic epilepsy have a 4-6% risk, while children of both parents with genetic epilepsy have a 12-20% risk.^1^

Recent reports have highlighted the importance of genetic predisposition in epilepsy syndromes, as ILAE has altered the previous “idiopathic” category to “genetic” and has approved of genetic testing for patients and families affected by epileptic syndromes including X-linked infantile spasm, Dravet syndrome, Ohtahara syndrome, and early-onset absence epilepsy.^18^ Furthermore, the new approaches to sequence DNA is revealing specific gene defects and linking them to distinct clinical features of genetic epilepsies.^8^

**Figure 1:**
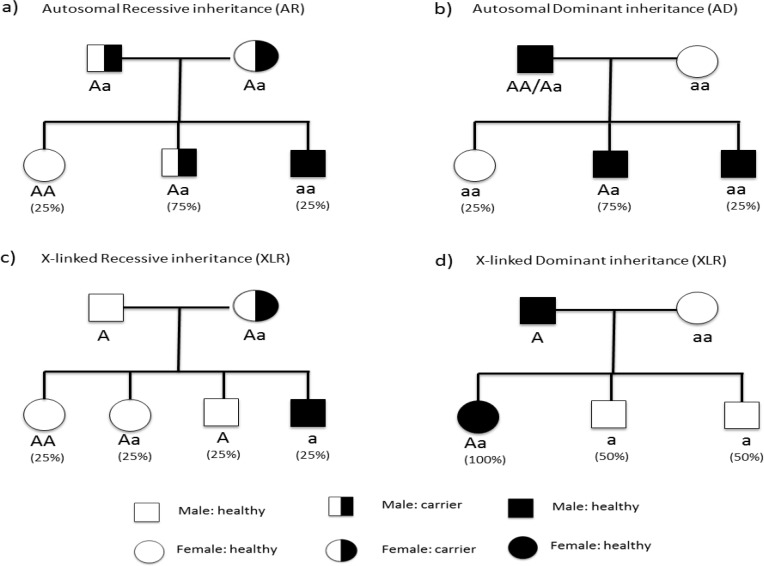
Mendelian modes of Inheritance (a) autosomal recessive inheritance. In this case “a” is the mutated allele of the gene and “A” is non-mutated. Individual who receives mutated allele from both parents (aa) would be affected with disease. Another persons “AA” and “Aa” do not show phenotypes of disease. (b) Autosomal dominant inheritance. In this model “A” (the dominant allele) is mutated allele and can cause disease, so any individual who receives just one mutated allele (AA, Aa) would be affected. (c) X-linked recessive inheritance. This mode has sex-based transmission because the gene is located on X chromosome, therefore females have two alleles of the gene and males have just one allele. If the mother is carrier, 50% of her boys will be affected and none of the girls in such pedigrees would show the phenotype of disease. (d) X-linked Dominant inheritance. In this example, the disease is caused by dominant mutated allele located in chromosome X. So if the father is affected, all the girls would be affected and no boys would show the disease phenotype. If the mother was affected too (Aa or AA) so the boys would have shown the phenotype of disease with different percentage

Genetic epilepsies could further divide into four subgroups according to the mechanism of inheritance: (1) genetic epilepsy with Mendelian inheritance, (2) epilepsies with complex inheritance, (3) genetic epilepsies associated with cytogenetic abnormalities and (4) Mendelian disorders in which epilepsy is one of the manifestations. The former class is thought to account for a small number of epilepsies, and the disease occurrence could be tracked through generation. A proper pedigree analysis will affirm whether the phenotype is dominant or recessive, autosomal or X-linked (Figure 1).^11^ Epilepsies with complex inheritance are believed to be involved in 50% of epilepsies.^11^^,^^19^ Although familial aggregation is seen through generations, the mode of inheritance cannot easily be identified.

Detection of a specific chromosomal abnormality (either structural or numerical) would be categorized under genetic epilepsies with cytogenetic abnormalities.^11^ This subgroup is mostly associated with other neurological disorders and facial anomalies.

Mendelian disorders in which epilepsy is one of the manifestations indicate multisystem disorders with epilepsy as one of the characteristics. These syndromes include neurocutaneous and neurodegenerative disorders and a cluster of metabolic diseases.^11^ Thus, the genetic counselor might be able to asses these syndromes. However, such disorders could hardly be considered an epileptic disease since epilepsy occurs as a secondary symptom.

The following section is dedicated to reviewing the role of chromosomal abnormalities and gene mutations in etiology of epileptic syndromes with some examples. We are referring interested readers for a complete list of genes mutated in epilepsies to two reviews written by Garofalo et al.^20^ and Kaneko et al.^21^

*Chromosomal aberrations*

Chromosomal aberration is characterized by atypical number or structural abnormality of at least one chromosome that usually leads to genetic disorders. In numerical group, aneuploidy is usually due to abnormal gametogenesis in parents.^22^ Considering aneuploidy is accompanied with gaining or losing considerable amount of genetic materials, apart from sex chromosome disorders it is a fatal.^23^ However, there are few cases of live birth. These patients usually suffer from facial dysmorphisms and mental retardation as well as seizure.^24^^,^^25^ Conventional karyotyping could easily identify numerical chromosomal aberrations.^26^

There are several forms of structural chromosomal abnormalities including deletion, duplication and translocation of portion of a chromosome. These defects are not generally fatal, and newborns with structural abnormalities may have developmental delay and facial dysmorphism.^27^ Epilepsy is one of the widespread features in this group of anomalies.

Ring chromosomes detection might help discovering genetic epilepsy.^28^^-^^30^ A ring chromosome is usually formed through breakage of both ends of the chromosome and fusion of arms. Back et al. have reported a phenotypically normal woman with ring chromosome 20 who had two children suffering from mental retardation, behavioral disorder, and epilepsy.^31^ In addition, EEG of epileptic patients with ring chromosome 20 has a distinct feature of prolonged high-voltage slow waves and seizures are resistant to medications.^28^ Ring chromosome 14 has also been reported to be resistant to antiepileptic therapy.^32^ The onset of epilepsy in this chromosomal disorder is often during the first year, mental retardation would be a constant character and the majority of cases have dysmorphic facial features. EEG frequently reveals focal abnormality.^32^

Chromosome 6q deletion (Long arm of chromosome 6) and chromosome 22q duplication have been shown to be associated with dysmorphic facial abnormalities, mental retardation and epilepsy.^33^^,^^34^

As a result of high-resolution karyotyping, many epileptic seizures have been linked to chromosomal abnormalities.^35^^-^^37^ Aberrations such as microdeletions and microduplications (microchromosomal defects) that could not be detected by conventional karyology might be identified by molecular cytogenetic approaches including comparative genomic hybridization (CGH) array and multiplex ligation-dependent probe amplification (MLPA).^38^^-^^41^ Exploring the nature of the human genome with high repetitive DNA sequences lead to discovering recurrent rearrangements of regions in some chromosomes such as 15q and 16p that are involved in epilepsy could result in recurrent heritable microdeletions and microduplications.^42^

***Gene mutations***

In addition to chromosomal abnormalities, gene mutations also could be associated with epilepsy syndromes. A good example would be genes encoding ion channel subunits.^43^^,^^44^ Excitatory or inhibitory neurotransmitters in central nervous system^45^ have also been recognized in Mendelian forms of epilepsy^11^^,^^46^^,^^47^ and thus, following simple Mendelian mode of inheritance.^46^^,^^47^ Genetic counseling could help identifying these disorders through a prodigy and risk of disease could be estimated for the next generation.

CHRNA4 gene encodes neural acetylcholine receptor subunit α4.^48^ It was the first gene to be associated with epilepsy syndromes. Mutation in this gene has been linked to Autosomal dominant nocturnal frontal lobe epilepsy.^49^^,^^50^ KCNQ2 and KCNQ3 genes that encode voltage-gated potassium channels were identified in families affected with benign familial neonatal seizures.^51^^,^^52^

At least 37 genes for generalized myoclonic epilepsy and febrile seizures, 47 genes for symptomatic (structural/metabolic) epilepsy and 30 genes for epileptic encephalopathies have been recognized.^20^ In a recent study of pediatric patients affected with infantile spasms and Lennox-Gastaut syndrome, two forms of epileptic encephalopathies, and their parents, researchers found 329 de novo mutations.^53^ These mutations are significantly more prominent in genes sets regulated by fragile X protein. Mutation of fragile X protein has been extensively discussed in autism spectrum disorders as it is the most widespread single-gene cause of autism ^54^. Further genetic defects involved in epileptic encephalopathies include MTOR, GABRA1 and FLNA.^53^

Mutation in SCN1A, a gene encoding voltage-gated sodium channel, has been demonstrated to be involved in Dravet syndrome. The affected patients suffer from severe myoclonic epilepsy during infancy with poor prognosis, as seizures are frequent, prolonged and resistant to treatment. Developmental delay will appear and some would have cognitive impairment. There are reports of mutation in PCDH19, a gene that encodes a calcium-dependent cell-adhesion protein and is located on chromosome X, in female patients with clinical symptoms related to Dravet syndrome.^55^^,^^56^ Interestingly, 11-12% of affected patients, who did not show any mutation in the mentioned genes, had pathogenic copy number variations (CNVs) in SCN1A gene. These CNVs might be detected by array CGH and MLPA assay.^42^^,^^57^ Marini et al. showed that deletion of 9.3 Mb (49 genes) of chromosome 2q without harming SCN1A gene could also result in Dravert phenotype.^57^

The prevalence of Unvericht-lunderborg disease or Baltic myoclonic epilepsy, a rare inherited form of epilepsy with progressive myoclonus, is higher in some regions (e.g. Sweden). This disorder has been associated with mutation of CSTB, a gene encoding cystatin B protein responsible for reducing the activity of cathepsins enzymes (protease)^58^ and is inherited in an autosomal recessive (AR) pattern.^59^ Furthermore, different type of gene mutations including CHRNA4 gene (frontal lobe epilepsy) have been reported in different populations.^49^^,^^50^^,^^60^ It seems necessary to identify specific mutations in distinct population to provide better genetic counseling for epilepsy.^21^

There are disorders that although epilepsy is one of the symptoms, it is not the core sign. Some examples are discussed below.

Lafora body disease, a neurodegenerative disorder, is a fatal glycogen metabolism disorder with AR inheritance^61^^,^^62^ and has been linked to EMP2A gene mutation (Lafarin protein).^63^

Neuronal ceroid lipofuscinoses, a cluster of at least 8 neurodegenerative disorders, a result of lysosomal storage defects and excessive accumulation of lipopigments in brain and other tissues. It has an AR pattern of inheritance.^21^ CLN1 (PPT1) and CLN3 gene mutations are mainly responsible for different types of disease.^64^

**Figure 2:**
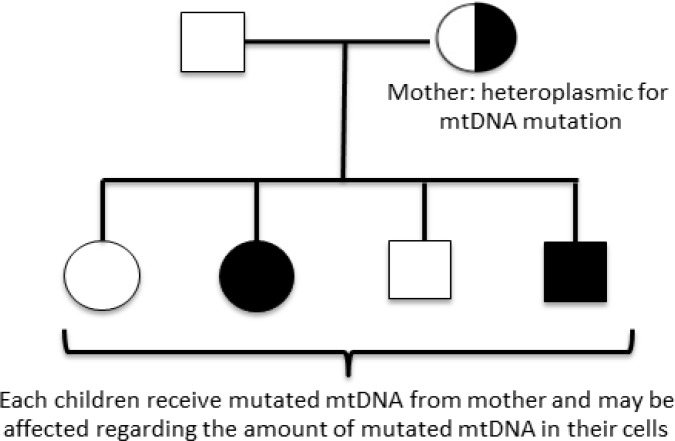
Transmission of mitochondrial DNA mutation in a hypothetical pedigree. This mode of inheritance is categorized as non-mendelian transmission because the mutated gene is not located in nuclear DNA. Mitochondria and its DNA (mtDNA) will transmit to next generation through oocyte cytoplasm so just mutated mtDNA from mother could cause the disease. Since, we have too many mitochondria and copies of mtDNA in a cell, the presence of disease and severity of its phenotype will be depended on the amount of mutated mtDNA inside individual’s cells. Heteroplasmy means both mutated and non-mutated mtDNA is present in a cell. Homoplasmy means the whole mtDNAs in a cell are mutated or non-mutated

Myoclonus epilepsy and ragged-red fibers are a rare mitochondrial disorder involving usually mutation of MT-TK gene located on mitochondrial DNA. It would lead to progressive neurological symptoms, including blindness and myoclonic epilepsy.^65^ Mitochondrial pattern of inheritance is relatively complex (Figure 2) as maternal mutated mitochondria affects zygote formation.^66^

Malformation of cortical development disorders represents a major spectrum of mental disabilities with severe epilepsies caused by defective neuronal migration. Mutation of LIS1 gene encoding microtubule-associated protein is one of the several genetic defects linked to these disorders. Lissencepahly with X-link gene mutation (xLIS)^67^ is another defective neuronal migration disorder that result in the lack of cerebral folds. Both genetic and non-genetic factors (e.g. viral infections of the fetus) are involved in etiology of these disorders.^68^

The introduction of new techniques of DNA sequencing has helped identifying point mutations, small insertions, and deletions.^38^

*Genetic counseling and genetic testing in epilepsy management*

Epilepsy is a multifactorial disease, and both genetic and environmental components are involved in etiology (Table 1). Various investigations, particularly twin studies, have contributed to detection the role of genetic elements in epilepsy syndromes. These findings will help predicting the clinical symptoms of the affected individual through genotype-phenotype correlation^69^ and conduct follow-up of high-risk pregnancies or an infant born in a family with increased rate of epilepsy.^70^ It will also aid the clinician in anticipating the clinical features in advance and manage in accordance.

Detection of specific genetic disorders will improve our understanding of the inheritance pattern. Thus, genetic counseling could better help families by estimating the risk of disease in next generation and family members of epileptic probands, who might be at greater risk for epilepsy syndromes.^69^ Interestingly, the same phenotype of epilepsy in different members of a pedigree could be due to different genetic defects.^67^ Consanguineous marriage will increase the risk of epilepsy syndrome, especially childhood onset of epilepsy^71^^-^^73^ and is a remarkable challenge for clinicians and geneticists in societies where it is a common tradition.

## Conclusion

We emphasize on cooperation of clinicians (particularly neurologist) and medical genetic experts in eastern societies like Iran, where consanguineous marriage is a common practice. This assistance is highlighted in high-risk families. It should be noted that prior to any genetic testing, patient and family members should be pre-tested in genetic counseling sessions.^18^

The genetic testing now commercially available for epilepsy includes analysis of 70 genes for detection of point mutations and deletion/duplications using DNA sequencing, CGH array, and MLPA techniques. The specimen used for genetic testing could be whole blood or any other body tissue appropriate for DNA extraction, for example, amniotic fluid, and chorionic villi samples are required for prenatal diagnosis.

Hence, collaboration of neurologist with geneticist in the case of genetic epilepsy will help the diagnosis and in some cases will improve management^20^.

**Table 1:** Summary of genetic abnormalities in different forms of epilepsies

**Epilepsy classification**	**Genetic abnormality**	**Genetic features**	**Example**	**Genetic test**
Epilepsy with mendelian inheritance	Specific gene mutation	Determined mode of inheritance	Generalized myoclonic epilepsy and febrile seizures	DNA sequencing, exom sequencing
Epilepsy with complex inheritance	Hard to find	Mode of inheritance could not be determined		Different tests
Epilepsy with chromosomal abnormality	Chromosomal aberrations	Usually sporadic	Ring chromosome 20, deletion of 6q, duplication 22q	karyotyping, CGH array, MLPA
Epilepsy associated with other mendelian disease	Specific gene mutation, mtDNA mutation	Determined mode of inheritance, sporadic	Lafora body disease, Neural ceroid lipofuscinoses, MERRF	DNA sequencing, exom sequencing

MERRF: Myoclonus epilepsy and ragged-red fibers; CGH: Comparative genomic hybridization; MLPA: Multiplex ligation-dependent probe amplification

## Notes

**How to cite this article: **Tafakhori A, Aghamollaii V, Faghihi-Kashani S, Sarraf P, Habibi L. Epileptic syndromes: From clinic to genetic. Iran J Neurol 2015; 14(1): 1-7.
